# Aromatic Profiles of Essential Oils from Five Commonly Used Thai Basils

**DOI:** 10.3390/foods7110175

**Published:** 2018-10-24

**Authors:** Tibet Tangpao, Hsiao-Hang Chung, Sarana Rose Sommano

**Affiliations:** 1 Major of Biotechnology, the Graduate School of Chiang Mai University, Chiang Mai 50200, Thailand; gr.zhk.88@gmail.com; 2 Plant Bioactive Compound Laboratory (BAC), Department of Plant and Soil Sciences, Faculty of Agriculture, Chiang Mai University, Chiang Mai 50200, Thailand; 3 Department of Horticulture, National Ilan University, Yilan City, Yilan County 260, Taiwan; hhchung@niu.edu.tw

**Keywords:** *Ocimum* spp., essential oil, aromatic profiles, Thai food

## Abstract

The research objectives of this study are to analyse the volatile compositions of different basil types available in Thai markets and to descriptively determine their aromatic qualities. Essential oils were hydro-distillated from fresh leaves of two Holy basil (*Ocimum sanctum*) varieties namely, white and red and other basil species, including Tree basil (*O. gratissimum*), Thai basil (*O. basilicum* var. thyrsiflorum), and Lemon basil (*O. citriodorum*). Oil physiochemical characteristics and volatile chromatograms from Gas Chromatography*–*Mass Spectrometry (GC-MS) were used to qualitatively and quantitatively describe the chemical compositions. Estragole, eugenol, and methyl eugenol were among the major volatiles found in the essential oils of these basil types. Classification by Principal Component Analysis (PCA) advised that these *Ocimum* spp. samples are grouped based on either the distinctive anise, citrus aroma (estragole, geranial and neral), or spice-like aroma (methyl eugenol, β-caryophyllene, and α-cubebene). The essential oils were also used for descriptive sensorial determination by five semi-trained panellists, using the following developed terms: anise, citrus, herb, spice, sweet, and woody. The panellists were able to differentiate essential oils of white Holy basil from red Holy basil based on the intensity of the anisic attribute, while the anise and citrus scents were detected as dominant in the Lemon basil, Tree basil, and Thai basil essential oils. The overall benefit from this research was the elucidation of aromatic qualities from Thai common *Ocimum* species in order to assess their potential as the raw materials for new food products.

## 1. Introduction

The genus *Ocimum* (belonging to the Lamiaceae which is recognised as the richest essential oil-bearing plant family) is represented by more than 150 species that are grown widely and distributed throughout tropical and temperate regions [1]. They are collectively known as the “basils” which are in commercial demand for their nutritional, aromatic, ornamental, culinary, religious, and medicinal importance [2]. Among those, Holy basil (*O. sanctum*), Sweet or Thai basil (*O. basilicum*), Lemon basil (*O. citriodorum*), and Tree basil (*O. gratissimum*) are frequently cultivated in several countries of South and South-East Asia including Thailand as culinary herbs [3,4]. There has been an increasing concern on liable health problems associated with synthetic food flavouring agents. Therefore, food researchers are focusing on the search for natural products that could replace chemically synthetised food additives [5,6].

Essential oil, as well as any other plant based natural ingredients, have been the beneficiary of legal, regulatory, and consumer preference as the result of a shared opinion on food safety [6,7]. Recent demands are for their essential oils, not only for medicinal industries but also for food industries [1,8,9,10]. Thus, it has come as no surprise that food suppliers of ethnic food ingredients quest for authentic Thai basils essential oil. However, the diversity of basil varieties, available as raw material for essential oil extraction, may lead to the confusion of basil aroma for Thai food. Varietal variations could also affect levels of volatile ingredients and profiles, thereby illustrating their distinctively sensorial aroma. Thai basils of different genotypes could be well differentiated on the basis of both essential oil compositions and sensory properties [11,12,13]. Within the genus *Ocimum*, the major constituents of essential oils are also diverse. From plants grown under the same condition, *O. basilicum* oil consisted of linalool with other constituents being camphor and humulene which gave the sweet-subtle aroma to its essential oil, while *O. sanctum* comprised a higher content of chavicol, eugenol, and eucalyptol [14]. None the less, there is limited data on aromatic profiles particularly of descriptive sensory analysis among five basil types available in Thailand viz., white and red Holy basil, Thai basil, Lemon basil, and Tree basil. The aim of this study is therefore to describe the aromatic identities of common Thai basils by chemical and sensory analyses.

## 2. Materials and Methods

### 2.1. Plant Material

To obtain reliable sources of material, the aerial parts of different stages were collected from five *Ocimum* spp. types viz., *O. sanctum* var. Rama (red), *O. sanctum* var. Shyama (white), *O. citriodorum*, *O. basilicum* var. thyrsiflorum, and *O. gratissimum* (100 plants/type) at their flowering stages. Plants were grown at Mae-Hia Agricultural Training and Research Centre, Division of Horticulture, Faculty of Agriculture, Chiang Mai University in an open-area (100 × 2500 cm plot with a plant spacing of 50 cm^2^) and maintained by watering for 2 h, 3 times per week using drip irrigation, and fertilising (Urea (46-0-0) and NPK fertiliser (15-15-15) in the ratio 1:3) once a month until flowering. After collection, plant specimens (leaf and floral specimens) were separated and their morphological appearances were recorded prior to sending them to the Department of Biology, Faculty of Science, Chiang Mai University for taxonomic confirmation.

### 2.2. Essential Oil Extraction

At the laboratory, leaf tissues were parted from the stems and inflorescence. Fresh leaves (~300 g) were used for essential oil extraction in a 5 L Clevenger hydro-distillation apparatus containing 2.5 L of distilled water and the extraction was for 2 h at 150 °C (MTopo^®^, heating mantle, Korea). After cooling to room temperature, the essential oil was collected and treated with anhydrous sodium sulphate to remove the remaining water. The yields were averaged from three separate extractions and calculated according to fresh weight of plant material [15].

### 2.3. Physical Characteristics of Essential Oil

Colour of the essential oil was determined by physical observation in day light and under ultraviolet radiation using an ultra-violet chamber [16]. The essential oil from water distillation was dissolved in methanol (10% *v*/*v*) and scanned for the absorbance with the wavelengths ranging from 220 to 500 nm by an UV-visible spectrophotometer (SPECTROstar Nano; BMG LABTECH, Offenburg, Germany) [17].

### 2.4. Gas Chromatography–Mass Spectrometry (GC-MS)

The Gas Chromatography–Mass Spectrometry (GC-MS) analyses were accomplished with a Bruker-scion 436 GC-MS equipped with 30 m × 0.25 mm**,** Rxi-5Sil MS column (Restek, Bellefonte, PA, USA). Essential oil samples (2 µL at the dilution of 1%, *v*/*v*, in dichloromethane with a presence of 0.003% *w*/*v* toluene as an internal standard) were injected in a split mode (1:20). The oven temperature was set at 60 °C for 3 min, and increased by 2.5 °C/min until 240 °C where it was held at this temperature for 10 min. The carrier gas was helium with a flow rate of 1.1 mL /min. The interface with MS was at 200 °C and mass spectra were taken at 70 eV in electron impact ionisation mode, with a scanning speed of 0.5 scans/s from *m*/*z* 20–350 [18]. The standard solution of C_8_–C_20_ n-alkane (Fluka^®^ Analytical, Munich, Germany) in hexane was also used for the calculation of retention indices (RI) [19]. The identification of the volatile compositions was by comparison with mass spectra in NIST 05.L and NIST 98.L libraries with >70% similarity. The compounds were confirmed by their RI as well as those from the literature [11]. The amount in µg/mL of essential oil was calculated as relative to that of internal standard.

### 2.5. Descriptive Analysis

Prior to sensory testing, five semi-trained panellists with extensive experience in Thai food (two males, three females, and age ranged from 25 to 37 years old) were chosen based on their abilities to discriminate differences and ranking/rating the intensity scents. They also completed a 3-day orientation and a 12 h descriptive training course. Thereafter, they then developed attribute terms describing the odour of five essential oils on cotton balls (200 µL). Panellists tasted all samples and discussed the attribute definitions, attribute references, reference intensities, and evaluation procedures. References were consistent with those of the previous studies as described in Table 1, although some additional terms (anise, herb, and woody) were added.

At the day of testing, the essential oil (200 µL oil pipetted on clean and deodourised cotton ball) and reference attributes were presented in front of the same group of the panellists. They then evaluated the intensity of the given attributes in triplicates on the 15-point interval scale (0 = none, 15 = extra strong) [20]. Clean air was obtained between each assessment. A gap of 20 s was sufficient to the individual odour assessments.

### 2.6. Statistical Analysis

Differences of the descriptive analysis data was determined using Analysis of Variance (ANOVA) by SPSS (IBM, Armonk, NY, USA) with a significance level of 0.05. Principal Component Analysis (PCA) was used to summarise graphical differences of volatile components of the essential oil and the descriptive data among the *Ocimum* spp. using XLSTAT ver. 2018.5 (New York, NY, USA).

## 3. Results and Discussion

### 3.1. Plant Identification and Physiochemical Characteristics of the Essential Oils

Basils are widely distributed in tropical areas and are likely to have originated in South Asia (India). These herbaceous plants are of annual type, usually propagated through seeds [4]. The genus of *Ocimum* comprises of more than 65 species and is the biggest genera in Lamiaceae family worldwide [24]. Different basil species can be identified by morphological characterisations such as leaf shape and its colour, flower structures and its colour, seed structures and its characteristics (Table 2). However, due to extensive cultivation, inter and intra-specific cross hybridisation has occurred leading to polyploidy and different numbers of species, subspecies, and varieties that are not significantly different in their appearances [24]. In our study, five *Ocimum* spp. types had distinct morphological characteristics. The *O. sanctum* of white and red varieties (viz., Rama and Shyama) possessed different leaf colours. *O. citriodorum* and *O. basilicum* var. thyrsiflorum illustrated unique seed characteristic which was mucilaginous after soaking in water. *O. gratissimum* possessed a large leaf size about 45 cm^2^ while *O. citriodorum* conferred leaf size around 3.5 cm^2^.

To obtain the essential oil, fresh basil leaves were extracted by hydro-distillation and the physiochemical characteristics were shown in Table 2. White Holy basil (*O. sanctum* var. Rama) and Thai basil (*O. basilicum* var. thyrsiflorum) illustrated maximum yield ~0.4%, Lemon basil (*O. citriodorum*) and red Holy basil (*O. sanctum* var. Shyama) yielded the essential oil of ~0.33%. However, Tree basil (*O. gratissimum*) gave the least essential oil content (<~0.2%). The colour (orange, yellow, and colourless) of essential oils under day light was different from species to species (*O. gratissimum*, *O. citriodorum*, *O. sanctum*, and *O. basilicum* var. thyrsiflorum) but not from variety to variety (*O. sanctum* var. Rama and Shyama). Variations in essential oil colours not only depends upon taxonomical characteristics but also relies on age of the plants as well as time of harvesting and different extraction techniques [25,26,27]. Siddique et al. [27] suggested that the chemical compositions of essential oil depend largely on its colour. Moreover, the alteration of the essential oil colour as a result of their compositions is suggested to be due to thermal degradation, oxidation, isomerisation, dehydrogenation and polymerisation [17,27,28,29].

**Figure 1:**
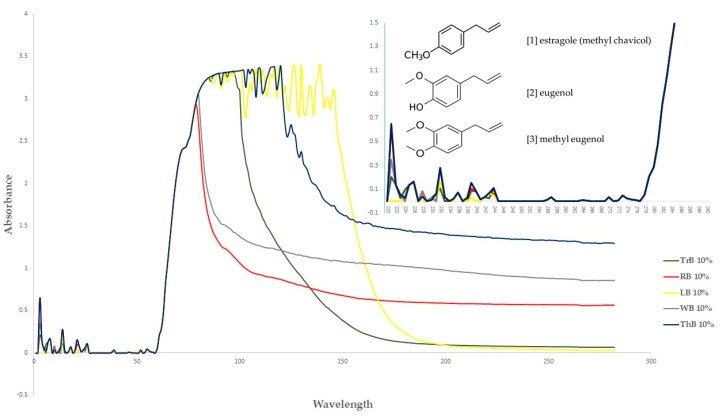
UV-Visible spectra of the essential oils from five *Ocimum* spp. The insertion is the inset evidences of the peaks between 220*–*280 nm and chemical structures of (1) estragole, (2) eugenol, (3) methyl eugenol. The essential oil was diluted in methanol. LB = Lemon basil (*O. citriodorum*); RB = red Holy basil (*O. sanctum* var. Shyama); ThB = Thai basil (*O. basilicum* var. thyrsiflorum); TrB = Tree basil (*O. gratissimum*); WB = white Holy basil (*O. sanctum* var. Rama).

Essential oils with different chemical compositions can absorb UV light at different wavelength therefore, illustrating variation of light reflection intensity [30,31]. In our experiment, white and red Holy basils essential oils reflected high UV light intensity, followed by moderate reflection intensity (Tree and Thai basils) and low reflection intensity (Lemon basil) (Table 2). These were confirmed by absorbance spectrum patterns under various UV-Visible wavelengths (220–500 nm) (Figure 1). The active chemical components of essential oil from *Ocimum* spp. plants are estragole (methyl chavicol), eugenol, and methyl eugenol [11,32,33]. Dighe et al. [34] advised that UV spectrum of eugenol gave the maximum absorption at 220*–*230 nm and a smaller peak at 278 nm which agree with our results. However, we found the tiny peaks at 269*–*275 instead of 278 nm (Figure 1).

### 3.2. Chemical Profile of the Essential Oil

The concentrations and the calculated retention indices of volatile compounds from the five *Ocimum* spp. essential oils are given in Table 3. Sixty-seven compounds were identified. The major components of essential oil from Lemon basil (*O. citriodorum*) volatile oil were estragole (methylchavicol) (98.22 µg/mL), citral (9.55 µg/mL), and neral (6.32 µg/mL). The principle components of red Holy basil (*O. sanctum* var. Shyama) were methyl eugenol (683.90 µg/mL), β-caryophyllene (145.81 µg/mL), and α-cubebene (104.70 µg/mL), and for Thai basil (*O. basilicum* var. thyrsiflorum) were estragole (methyl chavicol) (452.80 µg/mL), geranial (180.57 µg/mL), and neral (151.16 µg/mL). The main compositions of Tree basil (*O. gratissimum*) were eugenol (408.00 µg/mL), α-ocimene (256.45 µg/mL), and γ-muurolene (91.57 µg/mL) and in oil of white Holy basil (*O. sanctum* var. Rama) the major components were methyl eugenol (98.44 µg/mL), α-cubebene (9.94 µg/mL), and α-copaene (4.74 µg/mL).

From the literature, three major compounds were generally identified from the essential oil of *Ocimum* spp., viz. estragole (1), eugenol (2), and methyl eugenol (3) (Figure 1). Estragole (methyl chavicol) (1) is found in *O. basilicum* [11,13,35,36,37,38] and *O. citriodorum* [39]. Eugenol (2) is detected in *O. basilicum* [11,13], *O. utricifolium* [39], *O. sanctum* var. green and var. purple [14,40], *O. gratissimum* [38,41,42] essential oils. Methyl eugenol (3) is the principal composition in *O. sanctrm* var**.** Shyama [43], *O. uttricifolium* [44], *O. campechianum* Mill. [45] and some varieties of *O. basilicum* (i.e., var. dark green and var. purple opal) [12]. These results are in line with our work, however, the quantitative compositions of such chemicals may vary. Joshi and Si [46] and Murarikova et al. [12] described that variation in the qualitative and quantitative chemical profiles of the basil essential oil may be due to the variety of plant and their growing conditions such as season, climate or soil conditions. There was also some chemical variability of the essential oil of plants belonging to the Lamiacea family but within the same species, the major constituents can be similar [47,48]. In other plants, the factors being geographical differences and phenological stages of raw materials, such as in *Thymus algeriensis* [49] and *Hypericum* spp*.* [50].

**Figure 2:**
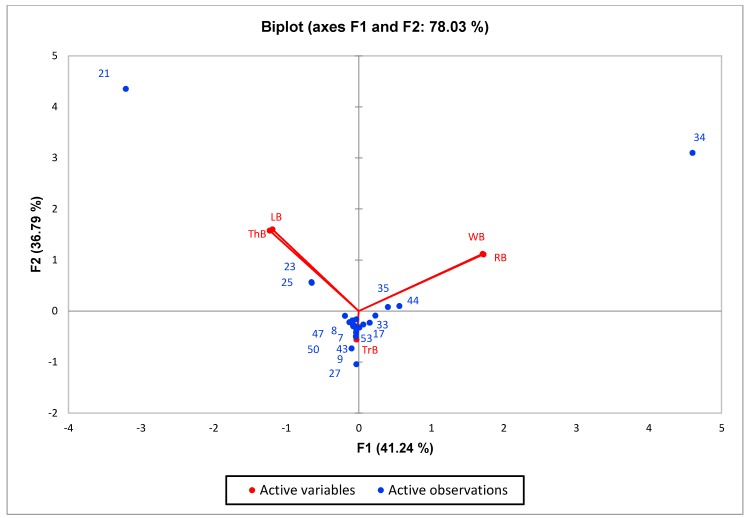
Principal Component Analysis (PCA) biplot illustrating the relationships among the chemical components and five *Ocimum* spp. Odour active compounds of 1*–*64 correspond to the code compounds in Table 3. LB = Lemon basil (*O. citriodorum*); RB = red Holy basil (*O. sanctum* var. Shyama); ThB = Thai basil (*O. basilicum* var. thyrsiflorum); TrB = Tree basil (*O. gratissimum*); WB = white Holy basil (*O. sanctum* var. Rama).

The PCA between the volatile compositions and the essential oil types revealed three major clustering groups (Figure 2). The first group included Thai basil and Lemon basil with the evident aromatic compounds (viz., estragole, geranial, and neral) which represent anise, lime-like, and fresh aroma [51]. The second cluster was of the white Holy basil and red Holy basil group with methyl eugenol, β-caryophyllene, and α-cubebene (herb and spice) as distinctive compounds [52]. White and red Holy basils are also variety related species (*O. sanctum*). The last group is the Tree basil that did not correlate with any groups described previously.

### 3.3. Sensory Profile of the Essential Oil

Overall sensory scores obtained by the different five *Ocimum* spp. essential oils are illustrated in Table 4. The intensity (0*–*15) represent the maximum possible aroma quantity of each identified attributes judged by the panelists. It was observed that the sweet attribute (scores ~2.4*–*3.4) was not significantly different among all types of the essential oils. The essential oils of Tree basil and Thai basil gave the highest intensity of herb odour (score ~9.0*–*11.0). Essential oils of white and red Holy basils provided the maximum woody scent (score ~2.2*–*3.4), while the Tree basil and white Holy basil dominated anisic attribute (score ~5.9*–*7.4). Tree basil and Thai basil oils illustrated the highest spice aroma intensity (score ~6.4*–*7.3). The citrus scent was the highest in the oil of Lemon basil with the score of 12.8.

**Figure 3:**
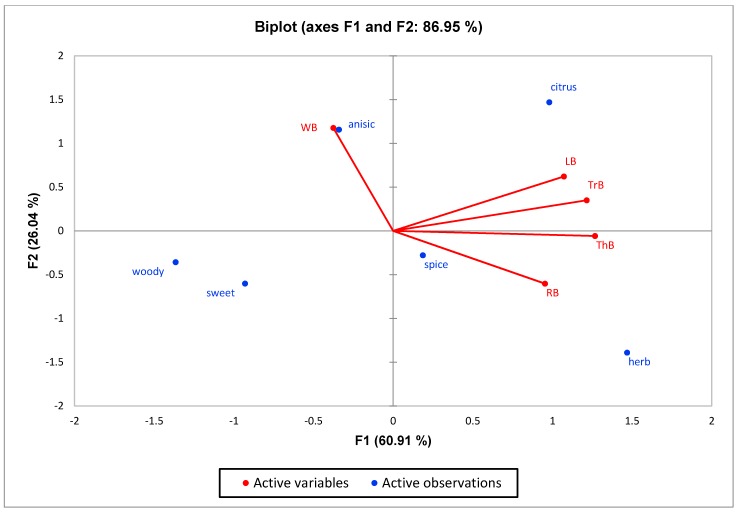
PCA biplot illustrating the relationships among the odour attributes and five *Ocimum* spp. LB = Lemon basil (*O. citriodorum*); RB = red Holy basil (*O. sanctum* var. Shyama); ThB = Thai basil (*O. basilicum* var. thyrsiflorum); TrB = Tree basil (*O. gratissimum*); WB = white Holy basil (*O. sanctum* var. Rama).

The PCA was able to split aromatic profile of white Holy basil relating with the higher intensity of anisic attribute (Figure 3). However, there was no correlation of woody and sweet attributes across the five essential oil types.

A study on the attribute of essential oil from *Ocimum* spp. (*O. basilicum* L.) by Calín-Sánchez et al. [53] evaluated the same attributes that are herbaceous (herb), spice, woody, and sweet. These attributes were also identified in our study. This group of researchers also found that the Sweet basil (*O. basilicum*) essential oil extracted from the dried leaves, gave stronger sweet and woody attributes than the essential oil extracted from fresh leaves. In our study, the citrus attribute is a principle characteristic of essential oils from most Thai basil types analyses especially the Lemon basil as confirmed by both chemical and sensory evaluations. This is consistent with the previous research by Al-Kateb and Mottram [54] who studied the relationship between growth stages and volatile compositions of Lemon basil (*O. citriodorum* Vis.). They found that citral, linalool, and estragole are major compounds of the essential oil and they contributed to the citrusy aroma.

## 4. Conclusions

From this research, we conclude that the major components of essential oils from the five types of basils used as food ingredients in Thailand are estragole, eugenol, and methyl eugenol. From this chemical analysis we can then distinguish *Ocimum* spp. plants from the odour characteristics into two groups: citrus and spice-like. Sensory analysis also confirmed that citrus is the main feature in these *Ocimum* spp. essential oils. It is also possible to sensorially separate the aromatic scent of the two Holy basil (var. red and white) essential oils by using anisic attributes. Future research could be extended to the use of the natural products from these plant species in food as additives active against food microbials and in agriculture (i.e., methyl eugenol) as bio-control agent.

## Figures and Tables

**Table 1:** Attributes and references used in evaluating five essential oil odour.

Odour Attributes	Reference Standard	*n*/15	Reference
**Anise**	anise powder, 2 g	10/15	*
**Citrus**	lemon extract (McCormick), 200 µL	8/15	[21]
**Herb**	thyme (McCormick), 0.5 g	10/15	*
**Spice**	ground allspice (McCormick), 0.5 g	8/15	[22]
**Sweet**	vanilla flavour (McCormick), 200 µL	10/15	[23]
**Woody**	peanut peel 2 g with 100 µL DI water	7/15	*

* Added terms from the panellists.

**Table 2:** Morphology of five *Ocimum* spp. and physiochemical characteristics of their essential oils.

Characteristics	LB	RB	ThB	TrB	WB
**Plant morphology**					
**General**	50–105 cm tall 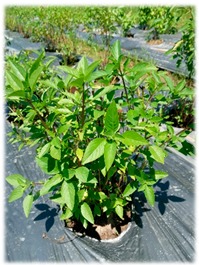	70–150 cm tall 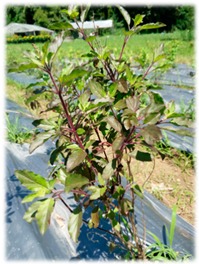	45–100 cm tall 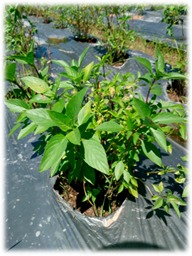	140–200 cm tall 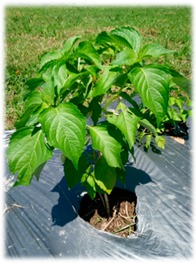	70–160 cm tall 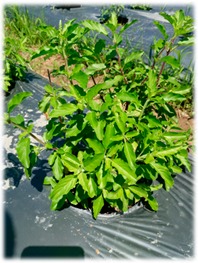
**Leaf structure**	leaf size ~3.5 × 1 cm, leaf elliptic-broadly obovate, glabrous except hairy midrib, veinlets and margin	leaf size ~4 × 1.5 cm, ovate-obovate, elliptic-oblong, surface patently hairy to clothed with soft spreading hair, Purple leaf	leaf size ~5.5 × 2 cm, leaf ovate-lanceolate to oblong-lanceolate, glabrous except hairy midrib, veinlets and margin	leaf size ~9 × 5 cm, leaf lanceolate, ovate or ovate-lanceolate, glabrous except hairy midrib	leaf size ~4 × 1.5 cm, leaf ovate-obovate, elliptic-oblong, surface patently hairy to clothed with soft spreading hair, green leaf
**Inflorescence** **and floral structure**	inflorescence greenish, flowers white, calyx green, long hairy	inflorescence purple, flowers purplish, calyx purple, patently hairy to densely pubescent	inflorescence greenish, flowers whitish pink, calyx green, long hairy	inflorescence greenish purple, flowers yellowish white, calyx greenish purple, hairy	inflorescence green-greenish purple, flowers purplish, calyx green, patently hairy to densely pubescent
**Seed characteristics**	seed brownish black, ellipsoid, mucilaginous	seed brown, globose, non-mucilaginous	seed brownish black, ellipsoid, mucilaginous	seed brown, subglobose, non-mucilaginous	seed brown, globose, non-mucilaginous
**Physiochemical Characteristics of essential oils**					
Yield *^,^**	0.37% ± 0.12 ^b^	0.33% ± 0.06 ^b^	0.43% ± 0.09 ^c^	0.19% ± 0.05 ^a^	0.47% ± 0.09 ^c^
**Colour** **under day light**	yellow	clear	yellow	orange	clear
**Colour** **under UV light** ***	+	+++	++	++	+++

LB = Lemon basil (*O. citriodorum*); RB = red Holy basil (*O. sanctum* var. Shyama); ThB = Thai basil (*O. basilicum* var. thyrsiflorum); TrB = Tree basil (*O. gratissimum*); WB = white Holy basil *(O. sanctum* var. Rama); * Values are mean ± SE (standard error) of 3 replications. ** Values followed by the different superscript letters (a–c) are significantly different at *p* = 0.05. *** + = degree of UV light reflection intensity.

**Table 3:** Chemical compositions of the essential oils from five *Ocimum* spp. plants.

No.	Compounds	Retention Index	Retention Index *	Amount of Chemicals				
				(µg/mL Essential Oils) **				
				LB	RB	ThB	TrB	WB
1	methyl 2-methylbutanoate	-	-	nd	nd	nd	0.89	nd
2	3-hexen-1-ol	850	-	nd	nd	nd	0.79	nd
3	α-pinene	930	938	nd	nd	nd	nd	0.32
4	camphene	944	-	nd	nd	nd	nd	0.42
5	β-pinene	973	981	1.31	nd	2.58	nd	0.52
6	1-octen-3-ol	979	-	nd	nd	nd	2.77	nd
7	myrcene	989	995	nd	nd	nd	5.13	nd
8	1,8-cineole	1028	1034	2.82	nd	5.64	nd	nd
9	α-ocimene	1049	1046	nd	nd	13.0	257	nd
10	z-ocimene	1051	-	5.83	nd	nd	17.7	nd
11	γ-terpinene	1058	-	0.36	nd	2.39	nd	nd
12	3-carene	1101	-	nd	1.76	14.61	22.3	nd
13	linalool	1104	1097	1.12	nd	nd	nd	1.09
14	(4e,6z)-allo-ocimene	1132	-	nd	nd	nd	14.9	nd
15	d-camphor	1145	1144	1.34	nd	3.54	nd	nd
16	trans-chrysanthemal	1152	-	nd	nd	2.20	nd	nd
17	borneol	1168	-	nd	7.79	nd	nd	2.80
18	1,4-heptadiene, 3-methyl-	1169	-	nd	nd	5.54	nd	nd
19	terpinen-4-ol	1180	-	1.78	nd	15.4	nd	nd
20	cyclohexane, ethenyl-	1189	-	nd	nd	6.78	nd	nd
21	estragole	1211	1196	98.2	nd	453	nd	nd
22	(r)-α-pinene	1236	-	nd	nd	8.60	nd	nd
23	neral	1251	1238	6.32	nd	151	nd	nd
24	(+) -(−)-3-carene	1264	-	nd	nd	6.30	nd	nd
25	geranial	1282	1268	nd	nd	181	nd	nd
26	citral	1283	-	9.55	nd	nd	nd	nd
27	eugenol	1371	1361	nd	nd	nd	408	1.50
28	α-copaene	1379	-	nd	20.7	nd	16.1	4.74
29	β-bourbonene	1388	1383	nd	7.68	nd	1.88	nd
30	4-methylpyrazole	1389	-	nd	nd	1.82	nd	nd
31	n-butylpyrrole	1391	-	nd	nd	nd	nd	2.25
32	β-cubebene	1396	1389	nd	1.98	nd	7.11	nd
33	β-elemene	1396	1391	0.77	65.7	4.30	3.65	2.79
34	methyl eugenol	1409	1411	0.77	684	2.87	nd	98.4
35	β-caryophyllene	1424	1420	1.34	146	10.6	17.3	nd
36	α-bergamotene	1439	1437	1.00	nd	4.49	0.69	nd
37	(z,e)-α-farnesene	1442	-	nd	nd	nd	51.6	nd
38	α-guaiene	1443	1439	0.19	nd	nd	nd	nd
39	β-sesquiphellandrene	1447	-	nd	nd	nd	0.59	nd
40	α-humulene	1458	1454	3.42	13.4	6.88	3.36	1.42
41	bicyclo sesquiphellandrene	1468	-	2.43	nd	10.8	0.59	nd
42	germacrene d	1469	1482	2.26	0.55	14.3	0.89	1.23
43	γ-muurolene	1490	-	nd	nd	nd	91.6	nd
44	α-cubebene	1490	-	nd	105	nd	nd	9.94
45	bicyclo [3.1.1] hept-3-ene-spiro-2,4′-(1′,3′-dioxane), 7,7-dimethyl-	1493	-	nd	1.87	nd	nd	nd
46	bicyclogermacrene	1500	1497	0.81	nd	3.63	3.55	nd
47	β-gurjunene	1501	-	nd	6.36	nd	nd	nd
48	α-selinene	1505	-	nd	nd	nd	nd	0.40
49	α-bulnesene	1509	1506	1.06	nd	3.92	nd	nd
50	α-farnesene	1511	-	nd	nd	nd	42.3	nd
51	α-amorphene	1518	-	nd	nd	2.58	nd	nd
52	phenylethanolamine	1519	-	nd	nd	nd	0.69	nd
53	δ-cadinene	1525	1524	nd	8.12	nd	11.1	0.56
54	1-bromo-8-heptadecyne	1539	-	0.44	nd	nd	nd	nd
55	(z)-4-decen-1-ol	1539	-	nd	3.84	nd	nd	nd
56	(z)-α-bisabolene	1546	1544	nd	nd	11.1	nd	nd
57	eremophilene	1556	-	nd	2.52	nd	nd	nd
58	elemol	1557	-	nd	nd	nd	nd	0.23
59	4-ethylphenethylamine	1582	-	nd	nd	nd	0.59	nd
60	ethyl trichloroacetate	1582	-	nd	1.75	nd	nd	nd
61	benzofuran, 7-(2,4-dinitrophenoxy)-3-ethoxy-2,3-dihydro-2,2-dimethyl-	1590	-	nd	0.99	nd	nd	nd
62	1,3-diisopropyl-1,3-cyclopentadiene	1602	-	nd	nd	nd	0.99	nd
63	cadina-1,4-diene	1622	-	nd	nd	1.24	nd	nd
64	naphthalene, 1,2,3,4,4a,7-hexahydro-1,6-dimethyl-4-(1-methylethyl)-	1622	-	0.31	nd	nd	nd	nd
65	bromoacetonitrile	1650	-	nd	nd	nd	0.50	nd
66	α-muurolene	1663	-	nd	2.41	nd	nd	nd
67	β-bisabolene	1690	-	0.27	nd	nd	nd	nd

* Retention index [11]. ** Values are calculated as reference to the internal standard toluene (0.003% *w/v*); nd = not detected. LB = Lemon basil (*O. citriodorum*); RB = red Holy basil (*O. sanctum* var. Shyama); ThB = Thai basil (*O. basilicum* var. thyrsiflorum); TrB = Tree basil (*O. gratissimum*); WB = white Holy basil *(O. sanctum* var. Rama). Values are proportionate to the internal toluene standard (0.003%). The Limit of Detection (LOD) and Limit of Quantitation (LOQ) are calculated from ranges of toluene concentrations (0.0015–0.03%). LOD = 0.083 µg/mL and LOQ = 0.835 µg/mL.

**Table 4:** The mean intensity values of the six attributes for the five *Ocimum* spp. essential oils in descriptive sensory evaluation.

Basils	Sweet	Herb	Woody	Anise	Citrus	Spice
**LB**	3.4 ± 0.24 ^a^	7.2 ± 0.49 ^bc^	0.98 ± 0.02 ^a^	4.72 ± 0.70 ^bc^	12.8 ± 0.58 ^d^	5.2 ± 0.66 ^b^
**RB**	2.6 ± 0.51 ^a^	5.68 ± 1.14 ^b^	2.26 ± 0.55 ^bc^	3.6 ± 0.51 ^ab^	2.7 ± 0.37 ^a^	3.5 ± 0.67 ^a^
**ThB**	2.4 ± 0.24 ^a^	11.2 ± 0.86 ^d^	1.6 ± 0.33 ^ab^	2.34 ± 0.52 ^a^	10.62 ± 0.69 ^c^	6.4 ± 0.24 ^bc^
**TrB**	2.46 ± 0.63 ^a^	9.2 ± 0.80 ^cd^	1.8 ± 0.20 ^ab^	7.4 ± 0.40 ^d^	8.9 ± 0.56 ^b^	7.28 ± 0.42 ^c^
**WB**	2.42 ± 0.50 ^a^	1.1 ± 0.10 ^a^	3.34 ± 0.50 ^c^	5.96 ± 0.87 ^cd^	4.2 ± 0.37 ^a^	2.7 ± 0.44 ^a^

Mean scores (*n* = 5) for each attribute within a column with different superscript letters (a–d) are significantly different at *p* = 0.05 using Duncan’s multiple comparison test. LB = Lemon basil (*O. citriodorum*); RB = red Holy basil (*O. sanctum* var. Shyama); ThB = Thai basil (*O. basilicum* var. thyrsiflorum); TrB = Tree basil (*O. gratissimum*); WB = white Holy basil *(O. sanctum* var. Rama).
